# Crystal structure of *rac*-3-hy­droxy-2-(*p*-tol­yl)-2,3,3a,4,7,7a-hexa­hydro-1*H*-4,7-methano­isoindol-1-one

**DOI:** 10.1107/S2056989015001942

**Published:** 2015-02-04

**Authors:** Mehmet Aslantaş, Cumali Çelik, Ömer Çelik, Arzu Karayel

**Affiliations:** a Department of Physics, Faculty of Sciences and Arts, University of Kahramanmaras Sutcuimam, Avsar Campus 46100, Kahramanmaras, Turkey; b Yalova Community Collage, University of Yalova, 77200 Yalova, Turkey; c Science and Technology Application and Research Center, Dicle University, 21280 Diyarbakır, Turkey; d Department of Physics, Faculty of Sciences and Arts, Hitit University, 19030 Çorum, Turkey; e Department of Physics, Bilkent University, 06800 Ankara, Turkey

**Keywords:** crystal structure, methano­isoindol-1-one, methano­iso­indole-1,3-dione, O—H⋯O hydrogen bonds, C—H⋯π inter­actions

## Abstract

In the title compound, C_16_H_17_NO_2_, the cyclo­hexene ring adopts a boat conformation, and the five-membered rings have envelope conformations with the bridging atom as the flap. Their mean planes are oriented at a dihedral angle of 86.51 (7)°. The mol­ecular structure is stabilized by a short intra­molecular C—H⋯O contact. In the crystal, mol­ecules are linked by O—H⋯O hydrogen bonds forming chains propagating along [100]. The chains are linked by C—H⋯π inter­actions, forming slabs parallel to (001).

## Related literature   

For medical and pharmaceutical applications of chiral tricyclic compounds, see: Abel *et al.* (1996 ▸); Salvati *et al.* (2005 ▸). For the synthesis of the starting reagent, 2-(*p*-tol­yl)-3a,4,7,7a-tetra­hydro-1*H*-4,7-methano­iso­indole-1,3(2*H*)-dione, see: Andrade & Evilazio (2004 ▸). For the reduction reaction used to synthesise the title compound, see: Hubert *et al.* (1975 ▸). For the crystal structure of a similar compound, see: Takebayashi *et al.* (2010 ▸).
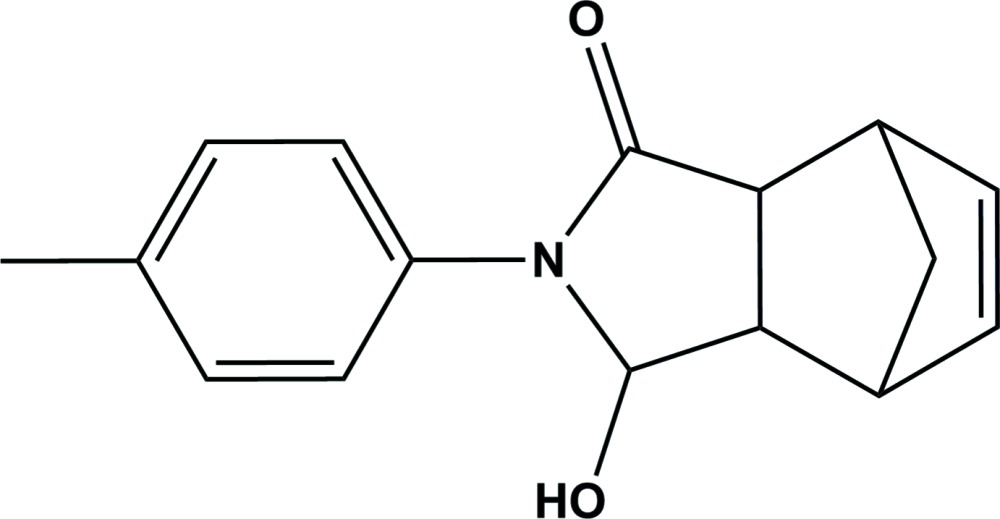


## Experimental   

### Crystal data  

C_16_H_17_NO_2_
*M* *_r_* = 255.31
Monoclinic, 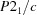

*a* = 6.5067 (2) Å
*b* = 9.7385 (2) Å
*c* = 21.0780 (5) Å
β = 97.154 (1)°
*V* = 1325.22 (6) Å^3^
*Z* = 4
Mo *K*α radiation
μ = 0.08 mm^−1^
*T* = 296 K
0.45 × 0.25 × 0.15 mm

### Data collection  

Bruker APEXII diffractometer
Absorption correction: multi-scan (Blessing, 1995 ▸) *T*_min_ = 0.963, *T*_max_ = 0.988
28760 measured reflections
5019 independent reflections
3930 reflections with *I* > 2σ(*I*)
*R*_int_ = 0.023

### Refinement  

*R*[*F* ^2^ > 2σ(*F* ^2^)] = 0.063
*wR*(*F* ^2^) = 0.180
*S* = 1.09
5019 reflections
180 parameters
H atoms treated by a mixture of independent and constrained refinement
Δρ_max_ = 0.40 e Å^−3^
Δρ_min_ = −0.38 e Å^−3^

### 

Data collection: *APEX2* (Bruker, 2007 ▸); cell refinement: *SAINT* (Bruker, 2007 ▸); data reduction: *SAINT*; program(s) used to solve structure: *SHELXS97* (Sheldrick, 2008 ▸); program(s) used to refine structure: *SHELXL97* (Sheldrick, 2015 ▸); molecular graphics: *ORTEP-3 for Windows* (Farrugia, 2012 ▸); software used to prepare material for publication: *WinGX* publication routines (Farrugia, 2012 ▸).

## Supplementary Material

Crystal structure: contains datablock(s) global, I. DOI: 10.1107/S2056989015001942/su5074sup1.cif

Structure factors: contains datablock(s) I. DOI: 10.1107/S2056989015001942/su5074Isup2.hkl

Supporting information file. DOI: 10.1107/S2056989015001942/su5074Isup3.cml

**Fig. 1.**
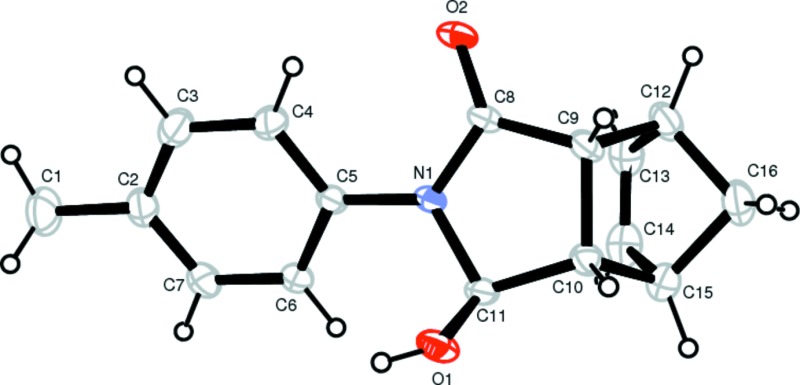
The molecular structure of the title compound, with atom labelling. Displacement ellipsoids are drawn at the 30% probability level.

**Fig. 2.**
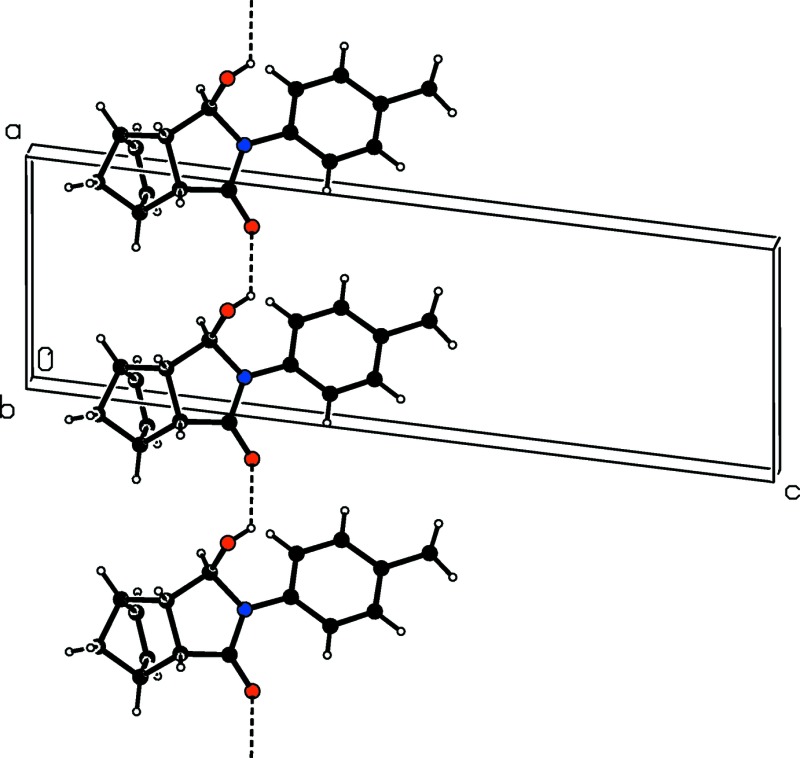
A partial view along the b axis of the crystal packing of the title compound. Dashed lines indicate the O—H···O hydrogen bonds (see Table 1 for details).

. DOI: 10.1107/S2056989015001942/su5074fig1.tif
The mol­ecular structure of the title compound, with atom labelling. Displacement ellipsoids are drawn at the 30% probability level.

b . DOI: 10.1107/S2056989015001942/su5074fig2.tif
A partial view along the *b* axis of the crystal packing of the title compound. Dashed lines indicate the O—H⋯O hydrogen bonds (see Table 1 for details).

CCDC reference: 1046290

Additional supporting information:  crystallographic information; 3D view; checkCIF report

## Figures and Tables

**Table 1:** Hydrogen-bond geometry (, ) *Cg*1 and *Cg*4 are the centroids of the N1/C8C11 and C2C7 rings, respectively.

*D*H*A*	*D*H	H*A*	*D* *A*	*D*H*A*
C4H4O2	0.93	2.33	2.860(2)	116
O1H2O2^i^	0.82	2.14	2.7194(15)	128
C13H13*Cg*1^ii^	0.93	2.94	3.6903(18)	139
C16H16*A* *Cg*4^iii^	0.99(2)	2.86(2)	3.692(2)	143.4(15)

Symmetry codes: (i) 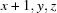; (ii) 
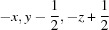
; (iii) 
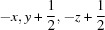
.

## supplementary crystallographic information

### S1. Comment

Chiral tricyclic compounds in heterocyclic chemistry are important in medicinal and pharmaceutical fields (Abel *et al.*, 1996; Salvati *et al.*, 2005). We report herein on the synthesis and crystal structure of the title compound, prepared by reduction of 2-(*p*-tolyl)-3*a*,4,7,7*a*-tetrahydro-1*H*-4,7-methanoisoindole-1,3(2*H*)-dione, using NaBH_4_.

The bond lengths and angles in the title compound, Fig. 1, are close to those reported for two similar chiral structures (Takebayashi *et al.*, 2010). The cyclohexene ring (C9/C190/C12-C15) has a normal boat conformation [puckering parameters: θ_2_ = 0.9587 (3) Å and φ_2_ = 169.02 (14)°]. The main bridge angle, C12—C16—C15, which connects the two bridgeheads on the cyclohexene ring, is 93.78 (12) °. The two five-membered rings, A(C9/C10/C15/C16/C12) and B(C12-C16) have envelope conformations with the flap atom C16 deviating from their mean planes by 0.5131 (2) and 0.4027 (2) Å, respectively. The dihedral angle between their mean planes, [A/B], is 86.51 (7)°. The whole molecule is non-planar with the dihedral angle between the benzene (C2-C7) and imide (N1/C8-C11) rings being 26.12 (5)°. This is much smaller than the same dihedral angle of ca.57.22 ° in the 2-phenyl derivative (Takebayashi *et al.*, 2010) or ca. 61.37 ° in the 2-(4-fluorophenyl) derivative (Takebayashi *et al.*, 2010). In the molecule there is a strong C—H···O intra-molecular contact present (Table 1).

In the crystal, molecules are linked by O—H···O hydrogen bonds forming chains along [100]; see Table 1 and Fig. 2. The chains are linked by C-H···π interactions forming slabs parallel to (001); see Table 1.

### S2. Experimental

The starting reagent, 2-(*p*-tolyl)-3*a*,4,7,7*a*-tetrahydro-1*H*-4,7-methanoisoindole-1,3(2*H*)-dione (*L*), is a known compound and was prepared from nadic anhydride and 4-toluidine (Andrade & Evilazio, 2004). The title compound was prepared by a reduction reaction following a modification of a literature procedure (Hubert *et al.*, 1975). NaBH_4_ (0.94 g) was added in small portions at 298 K over a period of 2 h to *L* (0.72 g, 2.84 mmol) dissolved in ethanol (250 ml). The excess of NaBH_4_ was consumed in 15 min at 278 K by adding aqueous HCl (2 mol dm^-3^) until the pH reached 3. The mixture was stirred for an additional 1 h at the same temperature then poured into water and extracted with dichloromethane. The organic layer was separated, dried over Na_2_SO_4_, filtered and evaporated to yield a white solid that was purified by silica gel chromatography [ethyl acetate/n-hexane (3:2 *v*/*v*)] which on slow evaporation of the solvent gave colourless crystals (yield: 65%; m.p.: 475–477 K). NMR (DMSO): δ(H) 1.38–1.42 (dd, 2H, CH_2_), 2.24 (s, 3H, CH_3_), 2.59–2.60 (d, H, CH), 2.61–2.62 (d, H, CH), 3.11–3.13 (m, H, CH), 3.18–3.21 (dd, H, CH), 4.81 (s, H, CH—OH), 6.03–6.05 (dd, H, ═CH), 6.16–6.18 (dd, H, ═CH), 7.09–7.11 (d, 2H, aromatic), 7.25–7.27 (d, 2H, aromatic); δ(C) 20.96 (CH_3_), 45.08 (CH), 45.62 (CH), 46.56 (CH), 49.58 (CH), 51.06 (CH_2_), 86.17 (CH—OH), 124.07 (*Cm*), 129.33 (Co), 134.37 (CH═CH), 134.93 (Cq—N), 135.87 (C—CH_3_), 174.39 (C═O) p.p.m.. FT—IR (ATR): 3211 (OH), 2972, 2943, 1646 (C═O), 1613 and 1515 (aromatic, C═C), 1422, 1403, 1065 (C—N), 819 cm^-1^.

### S3. Refinement

H atoms attached to bridging atom C16 were located in a difference Fourier map and freely refined. The other H atoms were placed in geometrically idealized positions (C—H = 0.93–0.98 Å and O—H= 0.82 Å) and treated as riding, with *U*_iso_(H) = 1.5*U*_eq_(O,C) for hydroxyl and methyl H atoms and = 1.2*U*_eq_(C) for other H atoms.

### Crystal data

**Table:**

C_16_H_17_NO_2_	*F*(000) = 544
*M**_r_* = 255.31	*D*_x_ = 1.280 Mg m^−^^3^
Monoclinic, *P*2_1_/*c*	Mo *K*α radiation, λ = 0.71073 Å
Hall symbol: -P 2ybc	Cell parameters from 5019 reflections
*a* = 6.5067 (2) Å	θ = 3.6–33.2°
*b* = 9.7385 (2) Å	µ = 0.08 mm^−^^1^
*c* = 21.0780 (5) Å	*T* = 296 K
β = 97.154 (1)°	Prism, colourless
*V* = 1325.22 (6) Å^3^	0.45 × 0.25 × 0.15 mm
*Z* = 4	

### Data collection

**Table:**

Bruker APEXII diffractometer	5019 independent reflections
Radiation source: fine-focus sealed tube	3930 reflections with *I* > 2σ(*I*)
Graphite monochromator	*R*_int_ = 0.023
φ and ω scans	θ_max_ = 33.2°, θ_min_ = 3.6°
Absorption correction: multi-scan (Blessing, 1995)	*h* = −5→9
*T*_min_ = 0.963, *T*_max_ = 0.988	*k* = −14→15
28760 measured reflections	*l* = −32→32

### Refinement

**Table:**

Refinement on *F*^2^	Primary atom site location: structure-invariant direct methods
Least-squares matrix: full	Secondary atom site location: difference Fourier map
*R*[*F*^2^ > 2σ(*F*^2^)] = 0.063	Hydrogen site location: inferred from neighbouring sites
*wR*(*F*^2^) = 0.180	H atoms treated by a mixture of independent and constrained refinement
*S* = 1.09	*w* = 1/[σ^2^(*F*_o_^2^) + (0.0762*P*)^2^ + 0.4345*P*] where *P* = (*F*_o_^2^ + 2*F*_c_^2^)/3
5019 reflections	(Δ/σ)_max_ < 0.001
180 parameters	Δρ_max_ = 0.40 e Å^−^^3^
0 restraints	Δρ_min_ = −0.38 e Å^−^^3^

### Special details

**Table:**

Geometry. All e.s.d.'s (except the e.s.d. in the dihedral angle between two l.s. planes) are estimated using the full covariance matrix. The cell e.s.d.'s are taken into account individually in the estimation of e.s.d.'s in distances, angles and torsion angles; correlations between e.s.d.'s in cell parameters are only used when they are defined by crystal symmetry. An approximate (isotropic) treatment of cell e.s.d.'s is used for estimating e.s.d.'s involving l.s. planes.
Refinement. Refinement of *F*^2^ against ALL reflections. The weighted *R*-factor *wR* and goodness of fit *S* are based on *F*^2^, conventional *R*-factors *R* are based on *F*, with *F* set to zero for negative *F*^2^. The threshold expression of *F*^2^ > σ(*F*^2^) is used only for calculating *R*-factors(gt) *etc*. and is not relevant to the choice of reflections for refinement. *R*-factors based on *F*^2^ are statistically about twice as large as those based on *F*, and *R*- factors based on ALL data will be even larger.

### Fractional atomic coordinates and isotropic or equivalent isotropic displacement parameters (Å^2^)

**Table:**

	*x*	*y*	*z*	*U*_iso_*/*U*_eq_	
O1	0.45122 (14)	1.12217 (12)	0.27036 (6)	0.0443 (3)	
H2	0.5256	1.0905	0.3011	0.066*	
O2	−0.18121 (15)	0.99108 (14)	0.30186 (6)	0.0498 (3)	
C16	−0.0698 (3)	1.0387 (2)	0.09543 (8)	0.0528 (4)	
C1	0.4966 (4)	0.7713 (3)	0.53749 (9)	0.0695 (6)	
H1A	0.4064	0.7943	0.5686	0.104*	
H1B	0.6307	0.8110	0.5498	0.104*	
H1C	0.5094	0.6733	0.5350	0.104*	
H16A	−0.073 (3)	1.138 (2)	0.0867 (10)	0.057 (6)*	
H16B	−0.110 (4)	0.990 (2)	0.0570 (12)	0.066 (6)*	
N1	0.16608 (15)	0.98524 (11)	0.29178 (5)	0.0303 (2)	
C11	0.30916 (17)	1.02207 (13)	0.24528 (6)	0.0323 (2)	
H11	0.3825	0.9402	0.2330	0.039*	
C5	0.24201 (17)	0.92979 (12)	0.35282 (6)	0.0303 (2)	
C8	−0.03527 (18)	1.01463 (13)	0.27142 (7)	0.0337 (2)	
C9	−0.05367 (19)	1.07927 (14)	0.20645 (7)	0.0363 (3)	
H9	−0.1068	1.1733	0.2075	0.044*	
C10	0.16740 (19)	1.07774 (14)	0.18773 (6)	0.0351 (3)	
H10	0.2096	1.1710	0.1777	0.042*	
C6	0.4272 (2)	0.85725 (15)	0.36000 (7)	0.0387 (3)	
H6	0.4973	0.8419	0.3248	0.046*	
C2	0.4079 (2)	0.82658 (16)	0.47322 (7)	0.0440 (3)	
C7	0.5078 (2)	0.80753 (17)	0.41974 (7)	0.0455 (3)	
H7	0.6327	0.7600	0.4239	0.055*	
C12	−0.1801 (2)	0.99380 (17)	0.15201 (8)	0.0470 (3)	
H12	−0.3307	1.0066	0.1470	0.056*	
C15	0.1436 (3)	0.98780 (17)	0.12617 (7)	0.0448 (3)	
H15	0.2563	0.9944	0.0995	0.054*	
C4	0.1392 (2)	0.94992 (18)	0.40597 (7)	0.0459 (3)	
H4	0.0148	0.9981	0.4021	0.055*	
C13	−0.1047 (3)	0.84754 (17)	0.16019 (8)	0.0530 (4)	
H13	−0.1791	0.7734	0.1733	0.064*	
C3	0.2228 (3)	0.8979 (2)	0.46493 (8)	0.0534 (4)	
H3	0.1517	0.9115	0.5001	0.064*	
C14	0.0870 (3)	0.84423 (17)	0.14533 (8)	0.0520 (4)	
H14	0.1720	0.7672	0.1465	0.062*	

### Atomic displacement parameters (Å^2^)

**Table:**

	*U*^11^	*U*^22^	*U*^33^	*U*^12^	*U*^13^	*U*^23^
O1	0.0241 (4)	0.0503 (6)	0.0575 (6)	−0.0066 (4)	0.0005 (4)	0.0026 (5)
O2	0.0203 (4)	0.0751 (8)	0.0549 (6)	0.0010 (4)	0.0079 (4)	0.0096 (5)
C16	0.0568 (9)	0.0558 (9)	0.0421 (8)	−0.0040 (8)	−0.0091 (7)	0.0114 (7)
C1	0.0762 (14)	0.0834 (14)	0.0453 (9)	−0.0020 (11)	−0.0064 (9)	0.0202 (9)
N1	0.0193 (4)	0.0366 (5)	0.0347 (5)	0.0025 (3)	0.0030 (3)	0.0021 (4)
C11	0.0218 (4)	0.0375 (6)	0.0380 (6)	0.0015 (4)	0.0058 (4)	0.0020 (5)
C5	0.0244 (5)	0.0332 (5)	0.0328 (5)	0.0007 (4)	0.0022 (4)	−0.0013 (4)
C8	0.0204 (4)	0.0377 (6)	0.0424 (6)	0.0021 (4)	0.0019 (4)	0.0008 (5)
C9	0.0254 (5)	0.0377 (6)	0.0444 (7)	0.0034 (4)	−0.0011 (4)	0.0061 (5)
C10	0.0300 (5)	0.0356 (6)	0.0395 (6)	−0.0011 (4)	0.0037 (4)	0.0071 (5)
C6	0.0287 (5)	0.0496 (7)	0.0379 (6)	0.0092 (5)	0.0050 (5)	0.0037 (5)
C2	0.0466 (7)	0.0473 (8)	0.0361 (6)	−0.0043 (6)	−0.0026 (5)	0.0045 (5)
C7	0.0358 (6)	0.0540 (8)	0.0450 (7)	0.0090 (6)	−0.0013 (5)	0.0087 (6)
C12	0.0351 (6)	0.0562 (9)	0.0464 (8)	−0.0055 (6)	−0.0089 (6)	0.0091 (6)
C15	0.0477 (8)	0.0496 (8)	0.0372 (7)	0.0010 (6)	0.0063 (6)	0.0052 (6)
C4	0.0422 (7)	0.0590 (9)	0.0377 (7)	0.0157 (6)	0.0093 (5)	−0.0018 (6)
C13	0.0641 (10)	0.0452 (8)	0.0459 (8)	−0.0178 (7)	−0.0079 (7)	0.0030 (6)
C3	0.0587 (9)	0.0672 (10)	0.0358 (7)	0.0103 (8)	0.0121 (6)	−0.0004 (7)
C14	0.0711 (11)	0.0396 (7)	0.0436 (8)	0.0024 (7)	−0.0003 (7)	−0.0026 (6)

### Geometric parameters (Å, º)

**Table:**

O1—C11	1.4008 (16)	C9—C12	1.566 (2)
O1—H2	0.8200	C9—H9	0.9800
O2—C8	1.2320 (16)	C10—C15	1.557 (2)
C16—C12	1.531 (2)	C10—H10	0.9800
C16—C15	1.539 (2)	C6—C7	1.3893 (19)
C16—H16A	0.99 (2)	C6—H6	0.9300
C16—H16B	0.95 (2)	C2—C7	1.382 (2)
C1—C2	1.505 (2)	C2—C3	1.383 (2)
C1—H1A	0.9600	C7—H7	0.9300
C1—H1B	0.9600	C12—C13	1.509 (3)
C1—H1C	0.9600	C12—H12	0.9800
N1—C8	1.3574 (14)	C15—C14	1.513 (2)
N1—C5	1.4256 (16)	C15—H15	0.9800
N1—C11	1.4776 (15)	C4—C3	1.389 (2)
C11—C10	1.5290 (18)	C4—H4	0.9300
C11—H11	0.9800	C13—C14	1.324 (3)
C5—C4	1.3884 (18)	C13—H13	0.9300
C5—C6	1.3885 (17)	C3—H3	0.9300
C8—C9	1.4984 (19)	C14—H14	0.9300
C9—C10	1.5383 (18)		
			
C11—O1—H2	109.5	C11—C10—H10	110.0
C12—C16—C15	93.78 (12)	C9—C10—H10	110.0
C12—C16—H16A	115.2 (12)	C15—C10—H10	110.0
C15—C16—H16A	113.0 (13)	C5—C6—C7	120.04 (13)
C12—C16—H16B	114.7 (15)	C5—C6—H6	120.0
C15—C16—H16B	109.9 (15)	C7—C6—H6	120.0
H16A—C16—H16B	109.4 (19)	C7—C2—C3	117.07 (13)
C2—C1—H1A	109.5	C7—C2—C1	121.32 (16)
C2—C1—H1B	109.5	C3—C2—C1	121.61 (16)
H1A—C1—H1B	109.5	C2—C7—C6	121.98 (13)
C2—C1—H1C	109.5	C2—C7—H7	119.0
H1A—C1—H1C	109.5	C6—C7—H7	119.0
H1B—C1—H1C	109.5	C13—C12—C16	100.44 (15)
C8—N1—C5	125.28 (10)	C13—C12—C9	106.51 (11)
C8—N1—C11	113.71 (10)	C16—C12—C9	99.46 (12)
C5—N1—C11	120.96 (9)	C13—C12—H12	116.0
O1—C11—N1	111.06 (11)	C16—C12—H12	116.0
O1—C11—C10	110.93 (11)	C9—C12—H12	116.0
N1—C11—C10	104.17 (9)	C14—C15—C16	99.98 (14)
O1—C11—H11	110.2	C14—C15—C10	107.43 (12)
N1—C11—H11	110.2	C16—C15—C10	99.24 (13)
C10—C11—H11	110.2	C14—C15—H15	115.9
C4—C5—C6	118.85 (12)	C16—C15—H15	115.9
C4—C5—N1	121.80 (11)	C10—C15—H15	115.9
C6—C5—N1	119.32 (11)	C5—C4—C3	119.75 (14)
O2—C8—N1	124.90 (13)	C5—C4—H4	120.1
O2—C8—C9	125.12 (11)	C3—C4—H4	120.1
N1—C8—C9	109.98 (11)	C14—C13—C12	107.37 (14)
C8—C9—C10	105.05 (10)	C14—C13—H13	126.3
C8—C9—C12	114.92 (11)	C12—C13—H13	126.3
C10—C9—C12	103.28 (12)	C2—C3—C4	122.30 (14)
C8—C9—H9	111.0	C2—C3—H3	118.9
C10—C9—H9	111.0	C4—C3—H3	118.9
C12—C9—H9	111.0	C13—C14—C15	107.95 (15)
C11—C10—C9	106.91 (10)	C13—C14—H14	126.0
C11—C10—C15	116.70 (11)	C15—C14—H14	126.0
C9—C10—C15	102.71 (11)		
			
C8—N1—C11—O1	117.43 (12)	C3—C2—C7—C6	−0.1 (3)
C5—N1—C11—O1	−60.01 (14)	C1—C2—C7—C6	179.60 (17)
C8—N1—C11—C10	−2.04 (14)	C5—C6—C7—C2	0.7 (2)
C5—N1—C11—C10	−179.48 (11)	C15—C16—C12—C13	−50.25 (14)
C8—N1—C5—C4	−26.3 (2)	C15—C16—C12—C9	58.64 (14)
C11—N1—C5—C4	150.86 (14)	C8—C9—C12—C13	−45.60 (17)
C8—N1—C5—C6	155.78 (13)	C10—C9—C12—C13	68.21 (15)
C11—N1—C5—C6	−27.09 (17)	C8—C9—C12—C16	−149.55 (12)
C5—N1—C8—O2	−3.7 (2)	C10—C9—C12—C16	−35.73 (14)
C11—N1—C8—O2	178.96 (13)	C12—C16—C15—C14	49.62 (15)
C5—N1—C8—C9	176.52 (11)	C12—C16—C15—C10	−60.06 (14)
C11—N1—C8—C9	−0.79 (15)	C11—C10—C15—C14	51.45 (17)
O2—C8—C9—C10	−176.50 (14)	C9—C10—C15—C14	−65.11 (15)
N1—C8—C9—C10	3.26 (15)	C11—C10—C15—C16	155.05 (12)
O2—C8—C9—C12	−63.72 (19)	C9—C10—C15—C16	38.49 (13)
N1—C8—C9—C12	116.04 (13)	C6—C5—C4—C3	0.1 (2)
O1—C11—C10—C9	−115.65 (11)	N1—C5—C4—C3	−177.82 (15)
N1—C11—C10—C9	3.91 (13)	C16—C12—C13—C14	33.93 (16)
O1—C11—C10—C15	130.13 (12)	C9—C12—C13—C14	−69.31 (17)
N1—C11—C10—C15	−110.31 (12)	C7—C2—C3—C4	−0.6 (3)
C8—C9—C10—C11	−4.38 (14)	C1—C2—C3—C4	179.80 (19)
C12—C9—C10—C11	−125.16 (11)	C5—C4—C3—C2	0.5 (3)
C8—C9—C10—C15	118.98 (12)	C12—C13—C14—C15	−0.61 (18)
C12—C9—C10—C15	−1.79 (13)	C16—C15—C14—C13	−32.66 (17)
C4—C5—C6—C7	−0.7 (2)	C10—C15—C14—C13	70.41 (17)
N1—C5—C6—C7	177.28 (13)		

### Hydrogen-bond geometry (Å, º)

Cg1 and Cg4 are the centroids of the N1/C8–C11 and C2–C7 rings, respectively.

**Table:**

*D*—H···*A*	*D*—H	H···*A*	*D*···*A*	*D*—H···*A*
C4—H4···O2	0.93	2.33	2.860 (2)	116
O1—H2···O2^i^	0.82	2.14	2.7194 (15)	128
C13—H13···*Cg*1^ii^	0.93	2.94	3.6903 (18)	139
C16—H16*A*···*Cg*4^iii^	0.99 (2)	2.86 (2)	3.692 (2)	143.4 (15)

Symmetry codes: (i) *x*+1, *y*, *z*; (ii) −*x*, *y*−1/2, −*z*+1/2; (iii) −*x*, *y*+1/2, −*z*+1/2.
