# Supplementary figures and images for: Crystal structure of rac-3-hy­droxy-2-(p-tol­yl)-2,3,3a,4,7,7a-hexa­hydro-1H-4,7-methano­isoindol-1-one
Source: Acta Crystallogr E Crystallogr Commun. 2015 Feb 4;71(Pt 3):o143–4. doi: 10.1107/S2056989015001942 (PMC4350749; doi:10.1107/S2056989015001942)

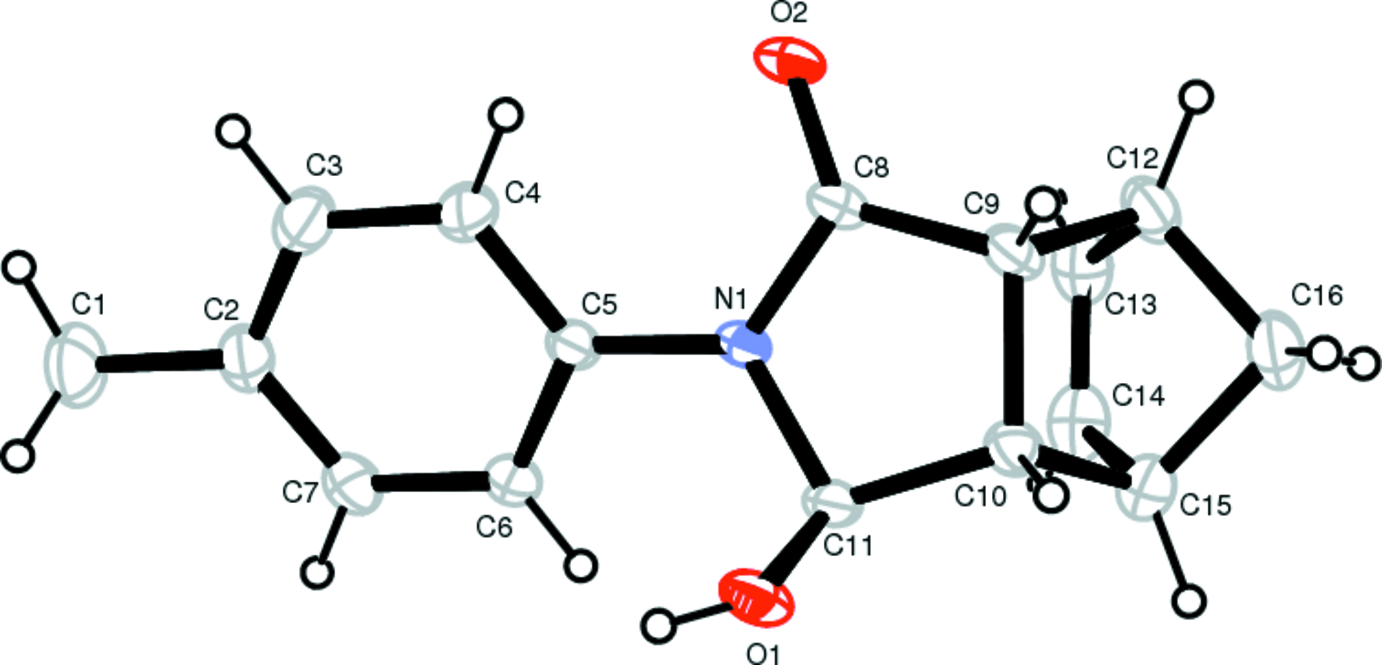

Supplement: Supplementary file 4 [file e-71-0o143-fig1.tif]

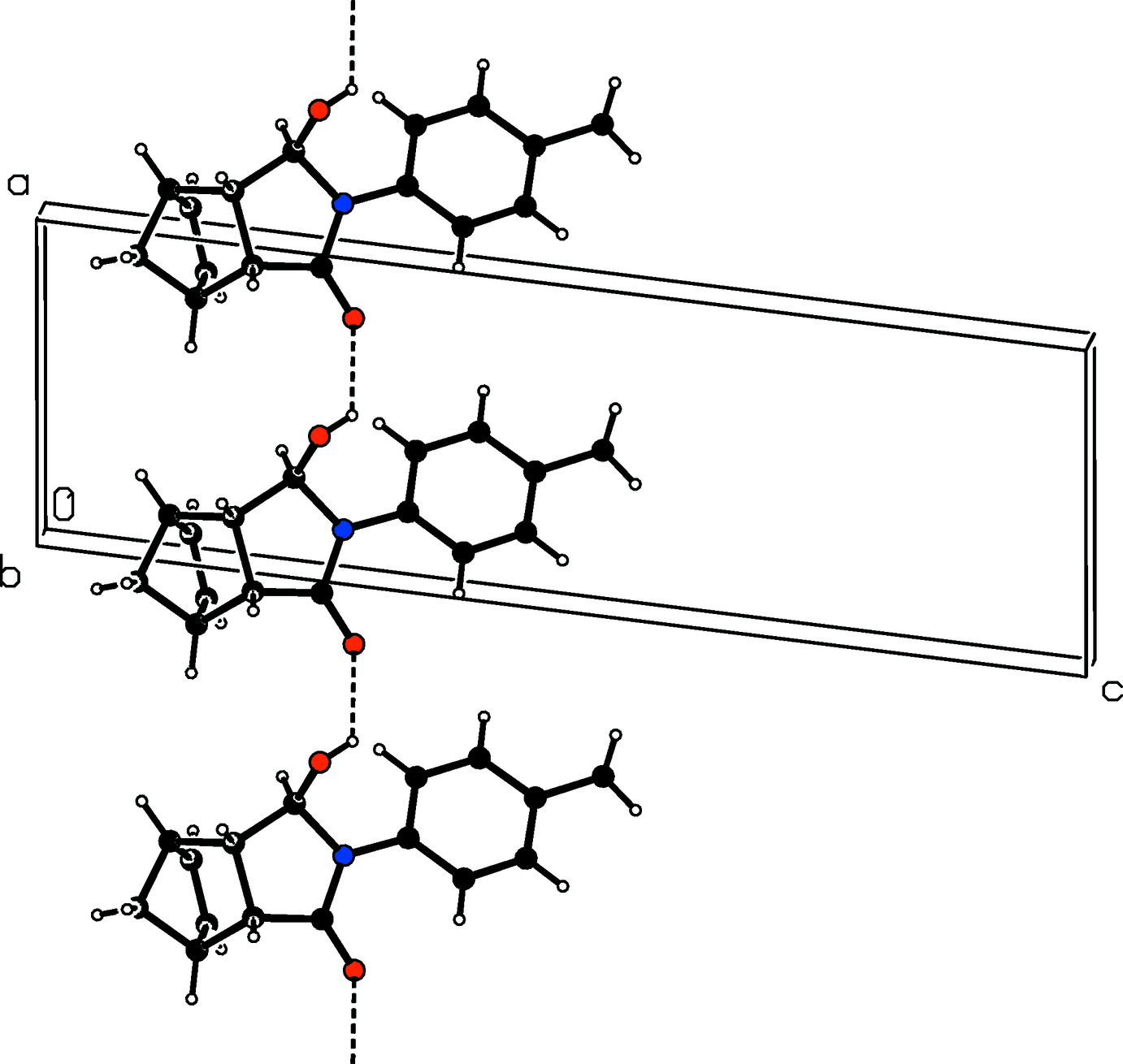

Supplement: Supplementary file 5 [file e-71-0o143-fig2.tif]
